# Exploring Chronic Pain, Immune Dysfunction and Lifestyle: A Focus on T Cell Exhaustion and Senescence

**DOI:** 10.3390/biom15111601

**Published:** 2025-11-15

**Authors:** Yanthe Buntinx, Jolien Hendrix, Arne Wyns, Jente Van Campenhout, Huan-Yu Xiong, Thessa Laeremans, Sara Cuesta-Sancho, Joeri L. Aerts, Jo Nijs, Andrea Polli

**Affiliations:** 1 Pain in Motion Research Group (PAIN), Department of Physiotherapy, Human Physiology and Anatomy, Faculty of Physical Education and Physiotherapy, Vrije Universiteit Brussel (VUB), Laarbeeklaan 103, 1090 Brussels, Belgium; yanthe.buntinx@vub.be (Y.B.); jolien.hendrix@vub.be (J.H.); arne.wyns@vub.be (A.W.); jente.van.campenhout@vub.be (J.V.C.); huanyu.xiong@vub.be (H.-Y.X.); jo.nijs@vub.be (J.N.); 2 Flanders Research Foundation-FWO, 1090 Brussels, Belgium; 3 Department of Public Health and Primary Care, Centre for Environment & Health, KU Leuven, Kapucijnenvoer 35, 3000 Leuven, Belgium; 4 Laboratory of Neuro-Aging & Viro-Immunotherapy, Center for Neurosciences, Vrije Universiteit Brussel (VUB), Laarbeeklaan 103, 1090 Brussels, Belgium; thessa.laeremans@vub.be (T.L.); joeri.aerts@vub.be (J.L.A.); 5 Mucosal Immunology Lab, Institute of Biomedicine and Molecular Genetics (IBGM), University of Valladolid-CSIC, 47005 Valladolid, Spain; sara.cuesta.uva@gmail.com; 6 Unit of Physiotherapy, Department of Health and Rehabilitation, Institute of Neuroscience and Physiology, Sahlgrenska Academy, University of Gothenburg, SE-405 30 Gothenburg, Sweden; 7 PijnPraxis.be Practice for Pain Management, 3970 Leopoldsburg, Belgium

**Keywords:** chronic pain, immune system, lifestyle factors, immune exhaustion, immune senescence, sleep, stress, physical activity, diet

## Abstract

Chronic pain conditions are debilitating and have an enormous impact on quality of life, yet underlying biological mechanisms remain poorly understood, hindering the development of diagnostic tools and effective treatments. Emerging evidence suggests a role for immune dysfunction in chronic pain. Among the various forms of immune dysfunction, T cell exhaustion and senescence, well-characterized in cancer and chronic infections, remain largely unexplored in chronic pain research. At the same time, lifestyle factors such as sleep, stress, physical activity, and diet are increasingly recognized as modulators of both pain and immune function. This review explores the potential interplay between these behavioural factors, immune exhaustion/senescence, and chronic pain. Critical gaps in current knowledge are identified, and future directions are outlined to clarify immune dysfunction and the influence of lifestyle factors in chronic pain conditions.

## 1. Linking Chronic Pain to Immune Dysfunction and Lifestyle Factors

Pain is the most common reason why individuals seek medical care and represents a significant burden on healthcare systems worldwide [1]. Chronic pain, defined as pain persisting for more than three months, is a multifaceted and debilitating condition affecting 11–40% of the population worldwide, with a higher prevalence in women [1,2,3]. Notably, three of the top four causes of disability (back pain, musculoskeletal disorders, and neck pain) are classified as chronic pain conditions [4]. Despite its profound impact on individuals, society, and the economy, chronic pain still lacks objective biomarkers for diagnosis [1]. This absence stems from a limited understanding of its underlying biological mechanisms, leaving diagnosis relying on subjective self-reporting [1,5]. The lack of biomarkers poses a barrier to the development of effective diagnostic tools and treatments for chronic pain, highlighting an urgent unmet medical need.

Mounting evidence supports the involvement of the immune system in chronic pain, with immune cells and nociceptors engaging in a dynamic, bidirectional network that modulates pain states [6,7]. Neuroimmune interactions are tightly regulated by both the central and peripheral nervous systems, for instance through stress-related pathways such as the hypothalamic–pituitary–adrenal (HPA) axis, which can influence immune cell activity and pain perception [8]. Despite evidence suggesting a role for immune dysfunction in chronic pain, focused research into immune states remains limited. Many studies are limited by small sample sizes and non-specific analyses of immune markers, leaving key questions unanswered [9]. Nonetheless, these findings are valuable when interpreted in the context of specific immune processes such as immune exhaustion and senescence, two distinct and fundamental states of the immune system. Exhaustion and senescence are particularly relevant in the context of chronic conditions. Unlike acute dysfunctional immune states, they represent long-term alterations of immune function [10]. Both processes are well-established contributors to pathologies such as cancer, chronic viral infections, and autoimmunity, where they have greatly advanced the understanding of immune dysfunction and therapeutic strategies [10,11,12]. In chronic pain, however, their contribution is remarkably underexplored.

Chronic pain is influenced by multiple lifestyle factors, amongst which are sleep, psychological stress, physical activity, and diet [13]. Each of these factors has independently been linked to both pain outcomes and immune regulation, which suggests an interesting interplay between chronic pain, lifestyle factors, and the immune system [14,15,16]. Dysregulation in these domains, such as sleep deprivation, chronic stress, and physical inactivity, can promote systemic low-grade inflammation and alter cytokine profiles [14,15,16]. These immune changes may, in turn, contribute to chronic pain through mechanisms such as neuroinflammation and central sensitization [6,17]. Investigating the influence of lifestyle factors may therefore offer valuable insights into the underlying biological contributors of chronic pain, particularly regarding immune-related mechanisms.

In this review, the potential interplay between the four main lifestyle factors (i.e., sleep, psychological stress, physical activity, and diet), immune exhaustion/senescence, and chronic pain is explored. Critical knowledge gaps are highlighted and directions for future research into immune processes and modifiable lifestyle factors in chronic pain conditions are proposed.

## 2. Immune Exhaustion and Senescence

The immune system plays a central role in defending the body against pathogens and injury through interactions between physical barriers, immune cells, and signalling molecules [18]. Immune dysfunction, however, is linked to a wide range of diseases, including cancer, autoimmune disorders, and neurodegenerative conditions [18,19,20]. Evidence also suggests a central role for immune dysfunction in chronic pain, where immune cells and nociceptors engage in bidirectional communication. Activated nociceptors release inflammatory mediators that further stimulate immune cells, sustaining a continuous feedback loop [7]. Moreover, immune signalling is implicated in the transition from acute to chronic pain, with T cells playing both protective and pain-promoting roles [9].

The activity of T cells is tightly regulated by stimulatory and inhibitory receptors on their surface, whose expression changes dynamically during differentiation [10]. Both CD4^+^ and CD8^+^ T cells interact with antigen-presenting cells (APCs) via these receptors, recognizing antigens presented on major histocompatibility complex (MHC) molecules [21]. Ligand binding modulates downstream signalling pathways, controlling cell activation and function [21]. The level of receptor expression thus finetunes the immune response and is used to identify specific T cell subsets, such as CD4^+^ and CD8^+^ T cells, and immune states. Among these, T cell exhaustion and T cell senescence are two immune states that share phenotypic similarities, including reduced functionality and altered expression of receptors, but differ fundamentally in their underlying triggers, molecular features, and reversibility.

Immune exhaustion refers to a progressive, yet potentially reversible, decline in T cell functionality, primarily affecting effector T cells [10]. Different from the regulatory suppression of T cells, the exhausted state is triggered by persistent antigenic overstimulation and prolonged immune activation, such as during cancer and chronic viral infections [10,11]. Exhausted T cells exhibit transcriptional and epigenetic changes, the gradual loss of effector function, a distinct cytokine profile, metabolic dysregulation, and overexpression of inhibitory receptors [22]. These include, but are not restricted to, programmed cell death protein 1 (PD-1), cytotoxic T-lymphocyte-associated antigen 4 (CTLA-4), B- and T-lymphocyte attenuator (BTLA), T-cell immunoglobulin and mucin domain 3 (Tim-3), T-cell immunoreceptor with Ig and ITIM domains (TIGIT), and lymphocyte-activation gene 3 (LAG-3) [22,23]. These receptors interact with their corresponding ligand(s) on APCs, such as PD-1 with programmed death-ligand 1 (PD-L1) and Tim-3 with Galectin-9 (Gal-9), thereby affecting T cell function [22,24].

At the transcriptional level, exhaustion is associated with dysregulation of key transcription factors involved in T cell differentiation, such as T-box expressed in T cells (T-bet), Eomesodermin (Eomes), and T cell factor 1 (TCF-1) [22,25]. Functionally, exhausted T cells show impaired production of cytotoxic effector proteins such as granzyme B and perforin, and reduced polyfunctional cytokine production of interleukin 2 (IL-2), interferon gamma (IFN-γ), and tumour necrosis factor alpha (TNF-α) [22,23,26]. Increased levels of IL-10 have also been implicated in promoting T cell exhaustion [23]. Importantly, exhaustion can be reversed, as evidenced by the remarkable clinical success of immune checkpoint inhibitors (ICIs) targeting PD-1 or CTLA-4, which reverse T cell exhaustion and are now established therapies for several cancers [27,28,29,30,31].

By contrast, immune senescence represents an irreversible state of cell cycle arrest; typically triggered by ageing or cellular stress [10,32]. While senescence plays beneficial roles in processes such as wound healing and tumour suppression, chronic senescence can lead to pathological outcomes [33]. Senescent immune cells exhibit telomere shortening and loss of telomerase activity, distinct transcriptional and epigenetic changes, metabolic dysregulation, apoptosis resistance, and a pro-inflammatory phenotype known as the senescence-associated secretory phenotype (SASP) [33,34,35,36]. SASP involves the release of signalling molecules, including IL-6, IL-10, TNF-α, and IFN-γ [36,37,38].

Senescent cells are further characterized by overexpression of surface markers, including Killer cell lectin-like receptor G1 (KLRG-1), Tim-3, TIGIT, and the terminal differentiation marker cluster of differentiation 57 (CD57), while having reduced CD27 and CD28 expression [10,22,36]. Unlike exhausted T cells, these cells have typically reached a terminal differentiation state and exhibit a reduced naive to memory ratio [36]. Functionally, senescent T cells show decreased granzyme B and perforin production, alongside a more pro-inflammatory secretory profile, with increased activity of cyclic GMP-AMP synthase (cGAS), nuclear factor kappa B (NF-κB), and cyclin-dependent kinase inhibitor 2A (p16^INK4a^), evoking SASP [34,38,39]. This shift toward increased cytokine-mediated signalling may contribute to sustained inflammation and sensitization, mechanisms which are increasingly recognized as relevant to chronic pain pathophysiology [8,9]. Immune senescence is irreversible; however, senolytic drugs have been developed to selectively target and remove senescent cells [40]. An overview of these properties, including the similarities and differences between T cell exhaustion and senescence, is summarized in Table 1.

Despite major advances in our understanding of immune exhaustion and senescence in the context of cancer and infectious diseases, where they have paved the way for therapy breakthroughs such as ICIs, their contribution to chronic pain remains largely unexplored [27,28,31]. This knowledge gap is striking and underscores the urgent need for in-depth studies focusing on T cell phenotypes in chronic pain populations.

## 3. Role of Lifestyle Factors in Immune Exhaustion and Senescence

**Figure 1:**
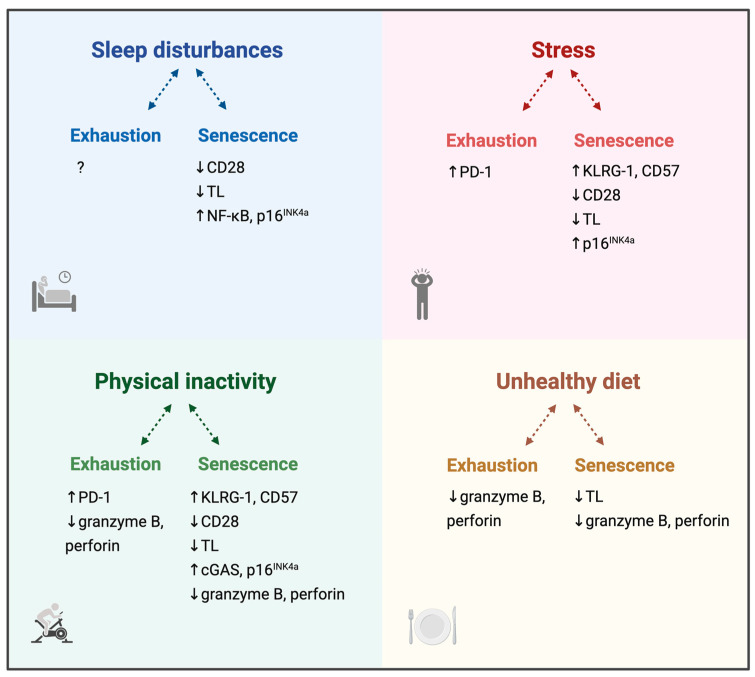
Associations between lifestyle factors and immune dysfunction interpreted in the context of T cell exhaustion and senescence. By looking at receptor expression, telomere length, effector proteins, and signalling molecules, signs of T cell exhaustion and senescence in human cohorts are summarized for sleep disturbances, psychological stress, physical activity, and healthy diet. Abbreviations: CD28/57 = cluster of differentiation 28/57; cGAS = cyclic GMP-AMP synthase; KLRG-1 = Killer cell lectin-like receptor G1; NF-κB = nuclear factor kappa B; PD-1 = programmed cell death protein 1; p16^INK4a^ = cyclin-dependent kinase inhibitor 2A; TL = telomere length; = increased; = decreased. Created in BioRender. Buntinx, Y. (2025) https://BioRender.com/hiv36in (accessed on 12 November 2025).

Sleep, stress, physical activity, and diet are considered the four main pillars of health and are essential for proper immune function [14,15,16]. When these lifestyle factors are not well-maintained or dysregulated, immune dysfunction can occur. Because they affect both immune function and chronic pain, exploring lifestyle factors could provide critical insights into how T cell exhaustion and senescence arise or persist in chronic pain. Current knowledge on the influence of sleep, psychological stress, physical activity, and diet, interpreted in the context of T cell exhaustion and senescence, is summarized in Figure 1. The next sections explore this information in more detail.

### 3.1. Sleep and T Cell Dysregulation

When interpreting current evidence of sleep and its effects on immune function in the context of T cell exhaustion and senescence, most studies remain limited and are restricted to either surface receptors analyses or functional assessments. Nevertheless, their insights are valuable. For instance, sleep fragmentation is correlated to an increase in late differentiated, CD8^+^CD28^−^ T cells, a hallmark of senescent T cells [41]. Yet, in mice, sleep fragmentation does not significantly alter PD-1 expression on CD4^+^ T cells, suggesting that sleep may not drive T cell exhaustion [42].

Sleep does not affect the number of granzyme B or perforin expressing cells [43]. However, sleep restriction has been shown to suppress T-bet expression, a key transcription factor regulating T cell differentiation and function [44]. This downregulation was accompanied by reduced IFN-γ levels and granzyme B expression [44]. Additionally, poor sleep quality is linked to elevated IL-6 production [45]. This effect is likely driven by activation of NF-κB, which regulates pro-inflammatory genes and was found to be increased in immune cells during sleep loss [44,46,47,48]. On the other hand, sleep deprivation also upregulates senescence-related genes such as p16^INK4a^, which normalize after successful remission of insomnia, a sleep-related disorder that is highly prevalent in the chronic pain population [47,49]. Most evidence on cytokine changes remains inconsistent, with studies reporting increased, decreased and unaffected cytokines such as IL-2, TNF-α, and IFN-γ [15].

Telomere shortening in relation to sleep has been examined in multiple studies, many of which indicate a correlation between poor sleep quality or duration and shorter telomere length (TL) in immune cells [50,51,52,53,54,55,56,57,58,59,60,61,62]. Prather et al. showed this association specifically in CD4^+^ and CD8^+^ T cells [52]. Interestingly, a recent study reported a genetic component for short sleep and its association with decreased TL [59]. However, not all studies confirm this relationship, with several reports of no significant association between sleep quality or duration and TL in immune cells [51,52,53,56,63,64,65]. Zgheib et al. linked sleep difficulties to shorter telomeres, although sleep duration itself was not significantly related [65]. Age plays an important role, as evidenced by Cribbet et al. who found an association between sleep and TL in older adults, but not in younger adults [53]. Other studies report sex-specific effects; Liang et al. in women under 50 years old, and Jackowska et al. in older men but not in women [54,55]. Several studies indicate that insomnia is associated with shorter telomeres, although results depend on age and are not always significant [66,67,68]. Conversely, one study found no relation between insomnia and TL [64]. For a more comprehensive overview of current knowledge on the link between sleep and TL, readers are referred to the review by Barragán et al. [69].

### 3.2. Stress and T Cell Dysregulation

Stress and stress-related disorders are linked to fewer naive and more terminally differentiated CD4^+^ and CD8^+^ T cells [70,71,72,73,74]. When looking at the expression of T cell surface markers, stress has been associated with upregulation of PD-1 and CD57 and downregulation of CTLA-4 and CD28 on CD4^+^ T cells, as well as increased KLRG-1, PD-1, and CD57, yet reduced CD28, expression on CD8^+^ T cells [70,75,76,77]. Duggal et al. strengthened these claims by combining T cell phenotyping with functional assays, demonstrating significant results between stress groups [75]. They also studied the effect of physical and psychological stress on T cells, showing that experiencing both stressors results in a higher senescent T cell phenotype than experiencing only one, indicating a link between stress and senescence [75]. Although conducted in a cancer context, one study showed that stress hormones modulate T cell phenotypes in humans and mice, influencing cytokine-mediated signalling and regulation of cell surface receptors, further supporting a potential link between stress and immune dysfunction [78]. Psychological stress is related to lower granzyme B and IFN-γ, as well as higher IL-10 production [79,80]. However, others report contradictory results by demonstrating elevated or conflicting levels of TNF-α, IFN-γ, IL-6, granzyme B, and perforin [43,75,80,81,82].

Ornish et al. demonstrated that lifestyle interventions, including stress management, physical activity, and dietary changes, are associated with longer TL, as measured by quantitative PCR (qPCR) [83]. This aligns with other studies linking stress to shorter telomeres [80,84,85,86,87]. Notably, while perceived stress is generally associated with shorter TL, this effect is not observed among individuals who are more physically active, suggesting a buffering effect of exercise [85]. Not all studies are consistent: one study reported no direct link between perceived stress and TL [52]. However, stress did influence the sleep–telomere relationship, with poor sleep linked to short telomeres in individuals with high perceived stress [52]. Rentscher et al. reported a link between stress and increased mRNA expression of p16^INK4a^, though no direct association with TL in leukocytes was observed [88]. Genetic susceptibility may further explain these associations, as individuals with the GABRA6 TT genotype, leading to increased stress susceptibility, have CD8^+^CD28^+^ T cells with shorter TL [89].

### 3.3. Physical Activity and T Cell Dysregulation

Exercise has been linked to lower expression of exhaustion and senescence markers (PD-1, KLRG-1, and CD57) and higher levels of CD28, granzyme B, and perforin [43,90,91,92,93]. Du et al. further showed that genes related to granzyme B, perforin, IL-2, and T-bet are upregulated in peripheral blood mononuclear cells (PBMCs) after acute exercise [94]. However, increased KLRG-1 and CD57 expression after exercise has also been demonstrated, while others found no effect on KLRG-1 expression on CD4^+^ T cells or the CD28^−^CD57^+^ phenotype [95,96,97,98]. Despite these inconsistencies, physically active individuals display a more favourable T cell phenotype than sedentary ones, suggesting that exercise has a moderating effect [95,96].

Physical activity has been linked to shifts in T cell composition, including a reduced naive to memory ratio, as well as conflicting findings on TNF-α, IFN-γ, and IL-2 production [90,93,96,99,100]. Exercise also reduces cGAS, p16^INK4a^, and other senescent-related proteins, potentially having a diminishing effect on immune senescence [90].

Most studies report a positive effect between physical activity and TL, with exercise being linked to longer TL [50,63,101,102,103,104,105,106,107,108,109,110]. Moreover, physical activity may also attenuate the effect of stress on TL, as their link has been described in sedentary individuals but not in those who are physically active [85]. However, evidence remains conflicting, as some studies indicate no effect of physical activity on TL [111,112,113].

### 3.4. Diet and T Cell Dysregulation

Human studies investigating the link between diet and T cell function are extremely limited, but preliminary data suggests that healthy nutrition correlates with a higher number of granzyme B and perforin expressing immune cells, showing a buffering effect of a healthy diet on immune dysfunction [43]. Animal studies could offer insights but remain scarce and highlight the need for more comprehensive research. In mice, a high-fat diet increased PD-1 expression on CD8^+^ T cells and reduced levels of granzyme B [114]. Caloric restriction in monkeys leads to an increase in naive T cells, decrease in memory T cells, and decrease in TNF-α and IFN-γ levels [115]. Another study, however, links caloric restriction to elevated IFN-γ protein and gene expression, without effect on IL-6 and IL-10 levels [116].

Research consistently shows that diet influences TL [117]. Consumption of foods such as nuts, vegetables, fruits, and coffee is linked to immune cells with longer telomeres [111,113,117,118,119,120]. On the other hand, the Western diet, characterized by higher fat and meat intake, has been associated with shorter TL [113,117,121].

### 3.5. Lifestyle Factors, Immune Exhaustion and Immune Senescence

There is growing evidence suggesting that poor sleep quality and duration are associated with altered immune function. However, when interpreted in the context of T cell exhaustion, current knowledge remains limited and inconsistent. While some studies report shorter telomeres and increased inflammatory signalling suggestive of immune senescence, others fail to confirm these in T cells specifically. Findings on cytokine profiles are particularly variable, highlighting the complexity of the relationship between sleep and T cell dysfunction. Importantly, assessing sleep is challenging as it can be subjective (e.g., using questionnaires) or objective (e.g., via polysomnography), complicating direct comparison between studies. In addition, sleep is closely related to other lifestyle factors such as psychological stress, with poor sleep reducing stress resilience and high stress levels leading to sleep disturbance.

Similarly, when investigating psychological stress and its influence on T cell differentiation and signalling, no clear conclusion on T cell exhaustion can be drawn. While stress has been associated with changes in exhaustion markers, such as altered cytokine levels (e.g., TNF-α, IFN-γ, and IL-10) and expression of T cell inhibitory receptors, these findings are limited and inconsistent across studies, which underscores the need for in-depth, targeted research. Substantial evidence links psychological stress to immune senescence, such as shorter telomeres, altered receptor expression, and elevated pro-inflammatory cytokines. However, as observed for sleep, evidence regarding cytokine levels is inconsistent, limiting direct comparison between studies. Future work should therefore combine both phenotypic markers and functional assays to better define the impact of stress on immune exhaustion and senescence, while considering other confounding lifestyle factors such as sleep, physical activity, and diet.

Similar to sleep and stress, physical activity is a broad and heterogeneous concept. An important distinction should be made between studies investigating acute exercise effects (following a single bout of exercise) and long-term training effects (resulting from regular exercise over weeks or months). This difference, along with factors such as timing of measurements, participants’ age, and type or intensity of exercise, all significantly influence outcomes. Through changes in stress hormones and energy metabolism, physical activity influences T cell activity and may therefore modulate exhaustion, but further research should investigate true T cell functionality to clarify its effect and resolve current inconsistencies [122]. Mounting evidence suggests that exercise attenuates the senescent phenotype, as it has been inversely associated with signs of senescence. In 2021, Barragán et al. and Mathot et al., respectively, reviewed how physical activity might modulate TL and other senescent markers; for more information readers are referred to their articles [69,123]. Current evidence suggests that long-term physical activity decreases T cell senescence in older adults.

Human studies on diet and immune regulation in the context of T cell differentiation, exhaustion, and senescence remain extremely limited. A healthy diet is associated with longer telomeres and higher frequencies of granzyme B and perforin-expressing immune cells, yet a clear conclusion on exhaustion or senescence cannot be drawn. Findings from animal studies further suggest that diet may influence immune senescence, but results remain inconsistent and are based on a limited number of models. Notably, metabolic diseases such as obesity and type 2 diabetes are linked to elevated pro-inflammatory cytokines and an accumulation of CD28^−^CD57^+^ T cells, reflecting immune senescence [124,125,126]. This aligns with the framework of this review, suggesting that dietary factors can shape T cell function and promote a chronic pro-inflammatory phenotype. However, the current lack of comprehensive experimental and human data highlights a key research gap, underscoring the need for well-controlled studies to clarify the causal effects of dietary effects on T cell exhaustion and senescence.

Overall, the interplay between lifestyle factors, immune exhaustion or senescence, and chronic pain is complex and characterized by conflicting findings. Current studies often investigate single variables and rarely control for confounding factors such as sleep, stress, physical activity, or diet simultaneously. For now, these associations should therefore be interpreted as correlative rather than causal. Future research integrating multiple lifestyle factors with in-depth immune phenotyping is essential to clarify causal relationships and their potential contribution to chronic pain.

## 4. Immune Exhaustion and Senescence in Chronic Pain Conditions

Despite substantial evidence implicating the involvement of the immune system in chronic pain, research into specific immune processes, such as exhaustion and senescence, remains limited [7,9]. Exploring T cell exhaustion and senescence in chronic pain conditions may therefore shed light on previously overlooked disease mechanisms.

### 4.1. Primary Chronic Pain Conditions

Chronic primary pain is defined as pain lasting for more than three months that is associated with significant functional disability and/or emotional distress, without being better explained by another condition [127]. Disorders such as myalgic encephalomyelitis/chronic fatigue syndrome (ME/CFS), fibromyalgia (FM), and chronic widespread pain (CWP) fall within this category and are characterized by persistent pain without a clear understanding of their underlying etiology and pathophysiology. In this context, T cell exhaustion and senescence may therefore not only arise as consequences of disease but could also contribute to the development or maintenance of chronic pain.

The potential link between ME/CFS and immune exhaustion or senescence has previously been reviewed [128]. This chronic disorder is often confused with macrophagic myofasciitis (MM), characterized by widespread pain and severe fatigue, or with long coronavirus disease (Long COVID). In MM, decreased TNF-α levels have been reported and interpreted as a reflection of an exhausted immune system as seen in ME/CFS [129]. Patients with Long COVID and ME/CFS symptoms exhibit elevated expression of exhaustion markers, including PD-1, Tim-3, TIGIT, and Gal-9 [130,131]. Saito et al. found that Tim-3^+^ cells correlate with impaired TNF-α and IFN-γ production, whereas PD-1^+^ cells are associated with elevated cytokines levels [130].

Several studies have studied cytokine levels in FM patients, with increased, decreased, and unchanged results for TNF-α, IFN-γ, IL-2, IL-6, and IL-10 [132,133,134,135,136,137,138,139,140,141,142]. However, these cytokines were measured in plasma or serum without confirmation by functional assays, such as in vitro T cell stimulation, limiting conclusions about T cell functionality. Wallace et al. investigated cytokine production in PBMCs, reporting no significant differences for TNF-α, IFN-γ, IL-2, and IL-10, and an increase for IL-6 [143]. In serum, elevated IL-6 levels correlate to FM symptoms and thus chronic pain, which may be suggestive of immune senescence [142].

A genomic study of patients with CWP identified differentially methylated regions in immune signalling pathways, including PD-1 and T cell exhaustion [144]. While preliminary, these findings suggest a potential role for immune exhaustion and highlight the need for more focused research.

### 4.2. Secondary Chronic Pain Conditions

In several disorders, chronic pain arises as a consequence of an underlying disease process, as is seen in cancer or rheumatoid arthritis (RA). In these contexts, specific pathophysiological mechanisms drive the development of chronic pain, and immune alterations such as T cell exhaustion and senescence might reflect disease progression or chronicity rather than act as primary drivers of chronic pain.

Genetic data suggests a possible link between exhaustion and RA, as T-bet gene polymorphisms have been associated with RA susceptibility [145]. Patients with RA also exhibit features of immune senescence, including telomere shortening, the CD28^−^CD57^+^ phenotype in CD4^+^ T cells, and elevated levels of TNF-α and IFN-γ [146,147,148,149]. While elevated levels of TNF-α and IFN-γ primarily reflect systemic inflammation, they may also indicate a role for senescent T cells in RA, as further described by Chalan et al. [150]. However, it remains unclear whether such immune changes directly contribute to chronic pain in RA.

In osteoarthritis (OA), a condition causing chronic pain in joints, decreased IL-10 levels have been linked to reduced Tim-3 expression, suggesting that T cell exhaustion is unlikely a prominent feature in this population [151]. However, pain-associated differently methylated regions in knee OA were enriched for genes involved in the PD-1 immunotherapy pathway, suggesting a potential role for T cell exhaustion [152]. Given the limited number of studies, interpreting these results in the context of immune exhaustion may be misleading.

Chronic osteomyelitis, a persistent bone infection accompanied by pain, has been associated with increased LAG-3 expression on T cells and reduced IFN-γ production, suggestive of T cell exhaustion [153]. In contrast, PD-1 and CD57 expression remained unchanged, and Tim-3 expression was reduced on T follicular helper cells [153,154]. Interestingly, B and NK cells of these patients show significant increases in the expression of exhaustion markers [154]. Moreover, a lack of alteration in CD57 expression suggests that T cell senescence is unlikely in people with osteomyelitis, although no strong conclusion can be drawn.

In chronic neuropathic pain, CD4^+^ T cells exhibit increased CD27 expression, contradicting a senescent phenotype [155]. Still, upregulation of NF-κB in T cells, and not in other immune cell types, alongside a reduction in CD8^+^ naive and an increase in CD4^+^ memory T cells, may reflect some sign of senescence [155]. Postherpetic neuralgia (PHN), a neuropathic pain condition following herpes zoster infection, has been linked to signs of T cell exhaustion. Patients with PHN show reduced CD28 expression in both CD4^+^ and CD8^+^ T cells, alongside increased levels of TNF-α, PD-1, CD57, and granzyme B [156]. Notably, T-bet is upregulated in patients’ CD4^+^ T cells but downregulated in their CD8^+^ T cells [156]. Furthermore, BTLA expression is positively correlated with pain scores, supporting a role for exhaustion in PHN [156].

Individuals suffering from sickle cell disease (SCD) have elevated levels of soluble exhaustion markers, including PD-1, BTLA, Tim-3, CTLA-4, and LAG-3, some of which highly correlate with pain [157]. However, elevated CD27 and CD28 levels in these patients contradict immune senescence [157]. It is important to note that pain in SCD is driven by well-established mechanisms such as inflammation and ischemia, which may overshadow contributions from T cell exhaustion or senescence [158]. Nonetheless, as there are currently no other SCD studies evaluating exhaustion and senescence, further research is warranted to clarify their potential role.

Taken together, these findings suggest that immune exhaustion features may occur in a variety of chronic pain conditions, both primary and secondary, although patterns remain condition-specific and inconsistent. While features of immune senescence are present in diseases like RA, they are not consistently observed across all chronic pain conditions. To clarify the role of immune exhaustion in chronic pain, standardized investigations of T cell phenotypes and cytokine profiles are needed, ideally in studies that carefully monitor or control for lifestyle factors known to affect immune states. Ultimately, future research should aim to clarify whether specific immune states, such as T cell exhaustion and senescence, contribute to chronic pain pathophysiology, and to what extent the established perpetuating role of an unhealthy lifestyle relates to these states in chronic pain. Additionally, while T cell subset-specific changes have been described in some chronic pain conditions, these are not always investigated in detail across all studies, making it difficult to draw in-depth conclusions on the role of subsets in T cell exhaustion or senescence. Integrating comprehensive subset analyses in future studies could provide a more complete understanding of immune dysregulation in chronic pain.

### 4.3. Potential Treatments

ICIs and senolytics, established therapies against exhaustion and senescence, respectively, illustrate how targeting dysfunctional immune states can lead to therapeutic breakthroughs. While their use is well-characterized in oncology, their application in chronic pain research is still in its very early stages. ICIs, drugs that target ligand-receptors interactions associated with exhaustion, modulate downstream signalling pathways and ultimately restore effective T cell function [40]. In the context of chronic pain, evidence is scarce. For instance, a recent study in cancer patients reported that PD-1/PD-L1 blockade on macrophages enhances chemotherapy-induced neuropathic pain [159]. This effect is likely driven by increased inflammation following checkpoint inhibition rather than direct reactivation of exhausted T cells, underscoring the complex interactions between immune checkpoints, immune cell function, and pain modulation. These findings highlight the need for dedicated research to investigate the role of T cell exhaustion, and its potential blockade, in chronic pain modulation. Additionally, the use of other ICIs, such as those targeting CTLA-4, should be studied, as preliminary evidence suggests PD-1-based ICIs may not be beneficial for chronic pain patients [160].

Senolytic drugs, on the other hand, do not reverse the senescent phenotype but instead cause selective cell death of senescent cells [161]. Recently, Mannarino et al. showed that senolytics diminish chronic low back pain in mice, although their study was not focused on T cells [162]. In line with this, the use of senolytics reduced neuropathic pain in mice and rat models [163,164,165]. Interestingly, senolytic drugs have already been tested in OA patients up until phase II clinical trials, moderately reducing associated pain levels [166,167]. However, senescence is an established feature in the chondrocytes of these patients, whereas its contribution to T cell function is not well understood.

## 5. Conclusions

Despite valuable insights discussed in this review, current studies on lifestyle factors and chronic pain are limited by incomplete T cell profiling and selective analysis of cytokines. Rather than focusing on a few selected markers, broader immune assessments using advanced techniques such as high-dimensional flow cytometry and single-cell RNA sequencing are needed. Moreover, the interplay between lifestyle factors and immune exhaustion or senescence remains complex, as most studies investigate these parameters in isolation and rarely account for potential confounders. Integrative, multi-variable approaches are therefore essential to unravel their complex interplay. In chronic pain research focusing on immunity, systematically incorporating lifestyle variables such as sleep, psychological stress, physical activity, and diet could provide important insights, as all these factors have been associated with both chronic pain and immune function, and may therefore act as modulators.

To achieve this, future studies should adopt more concrete and standardized approaches. Currently, the absence of standardized definitions and uniform objective measurements (e.g., receptor expression, cytokine profiles, and TL) complicates direct comparison across studies. Heterogeneity in study populations (e.g., age, sex, and comorbidities) and methodological approaches further complicate interpretation of current knowledge. Future studies should apply robust methodological designs, such as randomized cross-over designs, to examine causal interference. Importantly, interaction effects, such as how sleep, stress, physical activity, and diet may influence each other, TL, or ageing of the immune system, should always be considered. Finally, objective measurements should be combined with validated pain questionnaires to distinguish between immune alterations linked to disease mechanisms and those specifically associated with the experience of chronic pain, as these do not necessarily overlap.

Alongside methodological improvements, therapeutic implications should also be considered. Clarifying the therapeutic potential of ICIs and senolytics in chronic pain conditions represents an important future direction but requires careful characterization of T cell involvement before translation to patients.

## Figures and Tables

**Table 1:** Key characteristics of T cell exhaustion and senescence.

	T Cell Exhaustion	T Cell Senescence
**Trigger**	Persistent antigenic stimulation	Ageing, cellular stress
**Inhibitory receptors**	⇑ PD-1, CTLA-4, BTLA, Tim-3, TIGIT, Gal-9, LAG-3	⇑ CD57, KLRG-1, Tim-3, TIGIT
**Stimulatory receptors**	-	⇓ CD27, CD28
**Effector proteins, chemokines, and signalling molecules**	⇓ granzyme B, perforin	SASP ⇓ granzyme B, perforin ⇑ cGAS, NF-κB, p16^INK4a^
**Cytokines**	⇓ TNF-α, IFN-γ, IL-2 ⇑ IL-10	⇑ TNF-α, IFN-γ, IL-6, IL-10
**Transcription factors**	T-bet, Eomes, TCF-1	-
**Telomere length**	-	⇓
**Reversible?**	Yes	No

Abbreviations: BTLA = B- and T-lymphocyte attenuator; CD27 = cluster of differentiation 27; CD28 = cluster of differentiation 28; CD57 = cluster of differentiation 57; cGAS = cyclic GMP-AMP synthase; CTLA-4 = cytotoxic T-lymphocyte-associated antigen 4; Eomes = Eomesodermin; Gal-9 = galectin-9; IFN-γ = interferon gamma; IL-2 = interleukin 2; IL-6 = interleukin 6; IL-10 = interleukin 10; KLRG-1; Killer cell lectin-like receptor G1; LAG-3 = lymphocyte-activation gene 3; NF-κB = nuclear factor kappa B; PD-1 = programmed cell death protein 1; p16^INK4a^ = cyclin-dependent kinase inhibitor 2A; SASP = senescence-associated secretory phenotype; T-bet = T-box expressed in T cells; TCF-1 = T cell factor 1; TIGIT = T-cell immunoreceptor with Ig and ITIM domains; Tim-3 = T-cell immunoglobulin and mucin domain 3; TNF-α = tumour necrosis factor alpha; ⇑ = increased; ⇓ = decreased.

## Data Availability

No new data were created or analyzed in this study.

## Abbreviations

APCs	Antigen-presenting cells
BTLA	B- and T-lymphocyte attenuator
CD27/28/57	Cluster of differentiation 27/28/57
cGAS	Cyclic GMP-AMP synthase
COVID	Coronavirus disease
CTLA-4	Cytotoxic T-lymphocyte-associated antigen 4
CWP	Chronic widespread pain
Eomes	Eomesodermin
FM	Fibromyalgia
Gal-9	Galectin-9
HPA axis	Hypothalamic–pituitary–adrenal axis
ICIs	Immune checkpoint inhibitors
IFN-γ	Interferon gamma
IL-2/6/10	Interleukin 2/6/10
KLRG-1	Killer cell lectin-like receptor G1
LAG-3	Lymphocyte-activation gene 3
Long COVID	Long Coronavirus disease
MHC	Major histocompatibility complex
MM	Macrophagic myofasciitis
ME/CFS	Myalgic encephalomyelitis/chronic fatigue syndrome
NF-κB	Nuclear factor kappa B
NK cell	Natural killer cell
OA	Osteoarthritis
PBMCs	Peripheral blood mononuclear cells
PD-L1	Programmed Death-Ligand 1
PD-1	Programmed cell death protein 1
PHN	Postherpetic neuralgia
p16^INK4a^	Cyclin-dependent kinase inhibitor 2A
qPCR	Quantitative polymerase chain reaction
RA	Rheumatoid arthritis
SASP	Senescence-associated secretory phenotype
SCD	Sickle cell disease
T-bet	T-box expressed in T cells
TCF-1	T cell factor 1
TIGIT	T-cell immunoreceptor with Ig and ITIM domains
Tim-3	T-cell immunoglobulin and mucin domain 3
TL	Telomere length
TNF-α	Tumour necrosis factor alpha
